# Incorporation of Calcium Sulfate Dihydrate into a Mesoporous Calcium Silicate/Poly-ε-Caprolactone Scaffold to Regulate the Release of Bone Morphogenetic Protein-2 and Accelerate Bone Regeneration

**DOI:** 10.3390/biomedicines9020128

**Published:** 2021-01-29

**Authors:** Kuo-Hao Huang, Chen-Ying Wang, Cheng-Yu Chen, Tuan-Ti Hsu, Chun-Pin Lin

**Affiliations:** 1 Graduate Institute of Clinical Dentistry, School of Dentistry, National Taiwan University, Taipei 106319, Taiwan; d99422003@ntu.edu.tw (K.-H.H.); jybean@gmail.com (C.-Y.W.); k55599911@gmail.com (C.-Y.C.); 2 Department of Dentistry, National Taiwan University Hospital, Taipei 100229, Taiwan; 3 X-Dimension Center for Medical Research and Translation, China Medical University Hospital, Taichung 40447, Taiwan; 4 School of Dentistry, College of Dental Medicine, Kaohsiung Medical University, Kaohsiung 807378, Taiwan

**Keywords:** bone morphogenetic protein-2, calcium silicate, calcium sulfate, 3D printing, osteogenesis

## Abstract

Tissue engineering and scaffolds play an important role in tissue regeneration by supporting cell adhesion, proliferation, and differentiation. The design of a scaffold is critical in determining its feasibility, and it is critical to note that each tissue is unique in terms of its morphology and composition. However, calcium-silicate-based scaffolds are undegradable, which severely limits their application in bone regeneration. In this study, we developed a biodegradable mesoporous calcium silicate (MS)/calcium sulfate (CS)/poly-ε-caprolactone (PCL) composite and fabricated a composite scaffold with 3D printing technologies. In addition, we were able to load bone morphogenetic protein-2 (BMP-2) into MS powder via a one-step immersion procedure. The results demonstrated that the MS/CS scaffold gradually degraded within 3 months. More importantly, the scaffold exhibited a gradual release of BMP-2 throughout the test period. The adhesion and proliferation of human dental pulp stem cells on the MS/CS/BMP-2 (MS/CS/B) scaffold were significantly greater than that on the MS/CS scaffold. It was also found that cells cultured on the MS/CS/B scaffold had significantly higher levels of alkaline phosphatase activity and angiogenic-related protein expression. The MS/CS/B scaffold promoted the growth of new blood vessels and bone regeneration within 4 weeks of implantation in rabbits with induced critical-sized femoral defects. Therefore, it is hypothesized that the 3D-printed MS/CS/B scaffold can act both as a conventional BMP-2 delivery system and as an ideal osteoinductive biomaterial for bone regeneration.

## 1. Introduction

Bone defects are caused by surgery, fractures, tumors, or trauma and can affect people of all ages [1]. Typical healing of a fracture is characterized by four chronological phases: anabolic phase, inflammatory stage, endochondral stage, and remodeling phase of callus into bone [2]. Bone formation and regeneration are vital to the repair process. Current strategies for repairing bone defects include bone grafting, delivery of growth factors, and bioscaffolds [3]. The discovery of novel biomaterials is critical to tissue engineering, which has provided invaluable improvements in synthetic bone substitutes and advantages related to their use [4]. An advantage of these biomaterials is an extensive surface area, which allows for the active binding of native live bone tissues to the material. Furthermore, the evolution of regenerative medicine, coupled with 3D printing technologies, has enabled bone tissue engineering to become a potential solution for bone substitutes [5,6,7].

3D printing, or additive manufacturing, is a novel technology that combines biocompatible biomaterials for medical applications into a functional bioscaffold [8,9]. A 3D-printed scaffold allows cellular proliferation, differentiation, and repair of tissues or organs in an environment that is very similar to that of an in vivo host [10]. Previously, bone defects were repaired using bone graft techniques intended to augment bone formation through the recruitment of bone marrow stem cells, which then differentiate into osteoblasts at the injury site [11]. However, this process requires 6 months to 1 year if the defect is extensive. In addition to being too time consuming, another drawback is that it is expensive. In contrast, 3D printing can yield highly porous scaffolds that have been shown to allow rapid bone cell proliferation, differentiation, mineralization, and angiogenesis [12]. These 3D-printed scaffolds also double as a delivery system that transfers growth factors that motivate osteoinduction and osteoconduction [13]. More importantly, 3D printing allows for an individually customized product that can be broadly adapted for tissue engineering or clinical applications [14]. A 3D-printed polymeric scaffold incorporating hydrogel and primary cells can effectively repair a defect through complex patterns that direct cell migration, polarization, and organization and thus effectively enable tissue regeneration [15]. In addition, researchers have shown that 3D-printed calcium phosphate scaffolds with specific geometrical patterns promote osteoblast proliferation and enhance alkaline phosphatase (ALP) expression [16].

Researchers have since attempted to develop inorganic bone cements intended to overcome the limitations of traditional calcium phosphate cements [15]. Among these, calcium silicate cement is a commonly used inorganic bone cement used for bone tissue regeneration due to its excellent bioactivity, physicochemical properties, and biological properties [17,18]. In addition, numerous studies have proven the bioactivity, biocompatibility, biodegradability, and drug-loading capacity of calcium-silicate-based materials used for bone apatite formation and repair [19]. Calcium silicate is known to influence the proliferation and differentiation of several types of cells, such as periodontal ligament cells, pulp cells, and stem cells [20]. Furthermore, it has been reported that calcium silicate enhances the regeneration of hard tissue and periodontal tissue when used in combination with specific drugs [21]. Thus, calcium silicate is known to play a vital role in bone regeneration, particularly in bone remodeling and healing [22,23]. However, these materials are microsized and have a nano-pore structure, thus limiting their use in drug delivery and direct injection. Consequently, novel mesoporous calcium silicate (MS) nano-particles have been developed with diameters ranging between 2 and 50 nm, thus offering a greater surface area for the loading of materials [24].

Transforming growth factor-beta (TGF-β) and bone morphogenetic protein-2 (BMP-2) are critical osteoinductive growth factors [25]. Among all the US food and drug administration approved products, BMP-2 is one of the most commonly used proteins for bone regeneration and is one of the most widely studied members of the BMP family [26]. BMP-2 is used in bone regeneration for repairing bone defects, bone cell proliferation, and bone mineralization [17]. The controlled release of growth factors from scaffolds is a key factor in the repair of bone defects [27]. MS materials with prolonged drug release that also incorporate growth factors intended to extend the therapeutic effects of bone regeneration have been successfully designed in our laboratory. We combined MS with calcium sulfate (CS) to transport pharmaceuticals to their specified destination, after which they effectively degrade and rapidly release the drug at the targeted site. CS has been the most frequently used substitute for bone repair since 1982 due to its excellent bone regeneration and bone repair properties [26]. However, several disadvantages limit its fullest potential for clinical applications. For example, CS degrades at a faster rate than bones develop and thus creates pores in bones after the material has degraded [28].

In this study, we synthesized an inorganic biomaterial containing MS nano-particles and CS with the intention of effectively fabricating 3D-printed scaffolds. We assessed the physicochemical properties and cellular behavior of human dental pulp cells (hDPSCs) on these scaffolds and evaluated the feasibility of using the scaffolds as a carrier system for BMP-2. We also established rabbit bone defect models for the evaluation of bone formation in critically sized femoral defects. We strongly believe that the evidence in this study will provide helpful insights into scaffolds and their relationship with osteogenesis and angiogenesis. Furthermore, this study is intended to show the efficacy of MS/CS for bone regeneration.

## 2. Materials and Methods

### 2.1. Synthesis and Characterization of MS Nano-Particles

MS nano-particles were fabricated using our previously published protocols [17]. We mixed 3.3 g of cetyltrimethylammonium bromide (CTAB; Sigma-Aldrich, St. Louis, MO, USA) with 6 mL of ammonia (NH_3_) in 300 mL of double-distilled water and stirred the mixture for 30 min at 60 °C. Next, 15 mL of tetraethyl orthosilicate (Sigma-Aldrich) and 15.6 g of calcium nitrate were added, and the mixture was stirred for 3 h. Filtration was performed, and the precipitated products were washed thrice with 1 N of hydrochloric acid and ethanol. The washed products were dried overnight at 60 °C and further sintered at 800 °C for 2 h to remove remnants of CTAB. The mesoporous structure of the calcium silicate particles was characterized using transmission electron microscopy. We used field emission–scanning electron microscopy (FE-SEM; JEOL JSM-6700F, Tokyo, Japan) to examine the microstructure of the calcium silicate powder in 3 kV low secondary electrons image (LEI) mode. The phase composition of the powder was analyzed using X-ray diffractometry (XRD; Bruker D8SSS, Karlsruhe, Germany) at 30 kV and 30 mA, with a scanning speed of 1°/min.

### 2.2. Preparation of Mesoporous Calcium Silicate/Calcium Sulfate/Poly-ε-Caprolactone (MS/CS/PCL) Scaffolds

The thermal press method was used to fabricate the MS/CS/PCL matrix. CS (Sigma-Aldrich) and MS powders were placed in 100% alcohol, dripped into heated reagent-grade PCL (molecular weight = 43,000–50,000; Polysciences, Warrington, PA, USA), and dried in a dry-heat oven for another hour. 3D printing of the scaffolds was done using a Bio-Scaffolder 3.1 extrusion system (GeSiM, Grosserkmannsdorf, Germany). In brief, the MS/CS/PCL pastes were well mixed and dispensed through a steel nozzle at 80 °C with a pressure of 200–400 kPa. The mechanical and physical properties of the 3D-printed scaffolds were as follows: 7 × 500 µm lines (500 µm height) parallel to each other with a 500 µm gap in between, with 16 layers in total. After printing, the scaffolds were dried at room temperature for 2 h.

### 2.3. In Vitro Soaking

The simulated body fluid (SBF) solution used in this study had compounds similar to human blood plasma and consisted of 7.9949 g of NaCl, 0.2235 g of KCl, 0.147 g of K_2_HPO_4_, 0.3528 g of NaHCO_3_, 0.071 g of Na_2_SO_4_, 0.2775 g of CaCl_2_, and 0.305 g of MgCl_2_ in 6H_2_O, in 1000 mL of distilled H_2_O. The pH was adjusted to 7.4 with hydrochloric acid and tris(hydroxymethyl)aminomethane. After immersion for various durations (ranging from 1 day to 6 months), the scaffolds were removed from SBF and weighed on a laboratory scale (TE214S, Sartorius, Goettingen, Germany) to obtain the in vitro degradation profile. We used FE-SEM to examine the microstructure of the composite powder in 3 kV LEI mode. The mechanical properties of the scaffolds were evaluated using an EZ Test machine (Shimadzu, Kyoto, Japan) in accordance with our developed protocol. In addition, the recorded results were tabulated into a stress–strain curve. Six scaffolds were tested for each test, with the average recorded.

### 2.4. BMP-2 Loading

The scaffolds were immersed in 0.5 mg/mL BMP-2 (MP Biomedicals, Solon, OH, USA) solution for 12 h in order to load BMP-2 onto the surfaces of the scaffolds. The scaffolds were then centrifuged to remove excess BMP-2, washed, and lyophilized for 24 h. The scaffolds were then stored in a freezer in vacuum.

### 2.5. BMP-2 Release Kinetics

The BMP-2-loaded scaffolds were immersed in Dulbecco’s modified Eagle’s medium (DMEM) for this test. The DMEM was changed at specific time points and assessed for levels of BMP-2 using an enzyme-linked immunosorbent assay kit (Thermo Fisher Scientific, Carlsbad, CA, USA) and a spectrophotometer (TECAN Infinite Pro M200, Männedorf, Switzerland). All experiments were performed in triplicate, with the averages recorded.

### 2.6. Human Dental Pulp Stem Cell Culture and Expansion

In this study, we used human dental pulp stem cells (hDPSCs, PT-5025; Lonza, passages 3–8, Basel, Switzerland) to evaluate the biological properties of all 3D-printed scaffolds, and the cells were further cultured in a commercially available human dental pulp stem cell bullet kit (PT-3005; Lonza, Basel, Switzerland) under 5% CO_2_ at 37 °C. The hDPSCs grew on the tissue flask, in which the medium was changed every 2 days. In the osteogenic and angiogenic assay, the hDPSCs were measured using osteogenesis/angiogenesis assay kits (StemPro™ osteogenesis differentiation kit/angiogenesis starter kit; Invitrogen) for estimation of cell differentiation.

### 2.7. Cell Adhesion and Proliferation

Prior to cell cultures, all scaffolds were sterilized by immersion in 75% ethanol and 30 min of ultraviolet light exposure. The hDPSCs were trypsinized and pipetted into single cells (6 × 10^5^ cells/mL) in the medium and then seeded onto each scaffold. After culturing for various periods of time (ranging from 1 to 14 days), cell viability was evaluated using the PrestoBlue assay (Invitrogen) by detecting mitochondrial activity. Briefly, 15 µL of PrestoBlue solution and 145 µL of DMEM were added to each well and then incubated for 30 min. Thereafter, 150 µL of the solution from each well was transferred to a 96-well enzyme-linked immunosorbent assay plate. The plates were analyzed in a 96-well spectrophotometer at 570 nm with a reference wavelength of 600 nm. The hDPSCs cultured on culture plates were used as a control. The absorbance values were obtained in triplicate from three independent experiments for this assay.

### 2.8. Fluorescent Staining

To consider whether the BMP-2 released from the scaffold can affect cell attachment, we observed the cell morphology by staining after they were cultured on the scaffold for 3 and 7 days. Then, the scaffolds were washed with phosphate-buffered saline, fixed in 4% paraformaldehyde (Sigma-Aldrich) for 30 min, and then permeabilized with 0.1% Triton X-100 (Sigma-Aldrich) solution for 20 min at room temperature. The F-actin filaments were then stained with Alexa Fluor 488 phalloidin (1:400 dilution in phosphate-buffered saline (PBS); Invitrogen, Thermo Fisher Scientific) for 1 h. The nuclei were stained with 300 nM 4’,6-diamidino-2-phenylindole (DAPI; Invitrogen, Thermo Fisher Scientific) for 30 min. After washing the cells several times, the cell morphology was observed using a confocal laser scanning microscope (Leica TCS SP8; Wetzlar, Germany).

### 2.9. Osteogenic- and Angiogenic-Related Protein Secretion

After culturing hDPSCs on scaffolds for 7 days, alkaline phosphatase (ALP) was used to consider the early stage osteogenesis differentiation level of the hDPSCs. In brief, the hDPSCs were washed several times with cold PBS and measured for relative ALP activity by the p-nitrophenyl phosphate liquid substrate kit (Sigma) following the manufacturer’s instructions. In addition, the production of osteopontin (OPN), osteocalcin (OC), von Willebrand Factor (vWF), and angiopoietin 1 (Ang-1) proteins released from the cells was determined using an ELISA kit (Invitrogen), following the manufacturer’s instructions. The protein concentrations were measured based on correlations with a standard curve. All experiments were performed in triplicate.

### 2.10. Western Blot

After 3 days of culture, cells were lysed using Nonidet-P40 (NP40) buffer (Thermo), and a BCA protein assay kit was used to assess for the concentrations of the various proteins. Sodium dodecyl sulfate (SDS)–polyacrylamide gel electrophoresis was used to segregate the cell lysates (40 μg protein), which were then transferred onto polyvinylidene difluoride (PVDF) membranes (Merck Millipore, Burlington, MA, USA). These were placed in a solution containing 2% bovine serum albumin (BSA) and Tris-buffered saline with Tween 20 (TBST) for 1 h before they were exposed to primary anti-bone morphogenetic protein receptor type II (BMP2R), anti-phospho SMAD1/5/8, anti-phospho extracellular signal-regulated kinase 1/2 (p-ERK 1/2), anti-extracellular signal-regulated kinase 1/2 (ERK 1/2), anti-ALP, anti-OC, and β-actin (Abcam, Cambridge, MA, USA) for 2 h for further immunoblotting. Thereafter, horseradish peroxidase (HRP)-conjugated secondary antibodies were incubated with the samples for 1 h to enable chemiluminescence visualization. For this study, protein expression levels were normalized to β-actin. All experiments were performed in triplicate.

### 2.11. BMPR2 Knockdown Affecting Cell Viability and Differentiation

Validated select small interfering RNA (siRNA) was used to knock down the expression of bone morphogenetic protein receptor type II (BMPR2) (ID numbers: s2044; Life Technologies, Carlsbad, CA, USA). Transfections of the siRNA were performed using Lipofectamine^®^ 2000 Reagent (Invitrogen) according to the manufacturer’s protocol. BMPR2 protein expressions of the silenced proteins were then analyzed using a western blot. To further analyze the effects of BMPR2 silencing, the cells were assessed for subsequent cellular viability. All experiments were performed in triplicate.

### 2.12. Alizarin Red S Stain

Accumulated calcium deposition on the hDPSCs was analyzed using alizarin red S staining after 7, 10, and 14 days of culture on scaffolds in an osteogenic differentiation medium. Firstly, the specimens were fixed and stained with alizarin red S solution (pH = 4) for 20 min. After this, the scaffolds were washed several times and photographed using a BX53 Olympus fluorescence microscope (Olympus, Tokyo, Japan) at 200× magnification. In addition, the absorbance of the alizarin red S stain was quantified using a spectrophotometer at 450 nm in order to quantify our results. All experiments were performed in triplicate.

### 2.13. Rabbit Model of Femoral Bone Defects

The in vivo experimental protocol was approved by the Animal Experimental Ethics Committee of China Medical University in Taichung, Taiwan (CMUIACUC-2019-099-1). New Zealand white male rabbits aged 3 months and weighing 1.8–2.0 kg was purchased from the National Laboratory Animal Center (Taipei, Taiwan), and critically sized defects of the distal femoral epiphysis (6 mm in diameter, 6 mm in depth) were induced in them. The rabbits were divided into two groups of three rabbits each. The rabbits in one group were implanted with a scaffold containing BMP-2 at a concentration of 2 µg/mL, while the other group did not undergo implantation. The rabbits were first anesthetized with chlorhexidine for injection and then anesthetized continuously with 100% oxygen using a gas anesthesia machine (Engler ADS1000) with 5% isoflurane. Before the skin was dissected with a scalpel, the hair on the hind legs was shaved with an electric shaver and the skin was disinfected with alcohol and iodine. The muscle fascia was then dissected until the femur was exposed, taking care to avoid dissecting excess muscle and basic structures, such as nerves and blood vessels. The scaffold was implanted into the defect site, and the wound was closed with sutures and covered with a thick mask of anti-inflammatory ointment. All rabbits were fasted for 1 day before surgery.

### 2.14. Histological Staining

After 28 days, the samples were retrieved in accordance with protocols approved by our ethical committee. The samples were then washed, fixed, and sectioned at 6 mm a piece (OCT^®^) (KMA-0100-00A, CellPath Ltd., Newtown, Wales, UK). After this, a microtome was used to prepare 6 μm sections from each specimen. The sections were then stained with hematoxylin and eosin (H&E), a modified Masson’s trichrome stain kit (ScyTek Lab., West Logan, UT, USA), and a von Kossa kit (ScyTek). Trichrome staining in blue was used to identify collagen. Von Kossa staining in red was used to observe the difference between the osteoid tissue and the calcified bone. A BX53 Olympus microscope was used for the examination of the samples.

### 2.15. Statistical Analyses

A minimum of three independent tests was performed for each experiment, and all data in this study were reported as the mean ± standard deviation (SD). Student’s *t*-test was used to analyze the significant differences between groups. In all experiments, the results were considered statistically significant if the *p*-value was <0.05.

## 3. Results and Discussion

### 3.1. The Characterizations of MS and CS Materials

**Figure 1:**
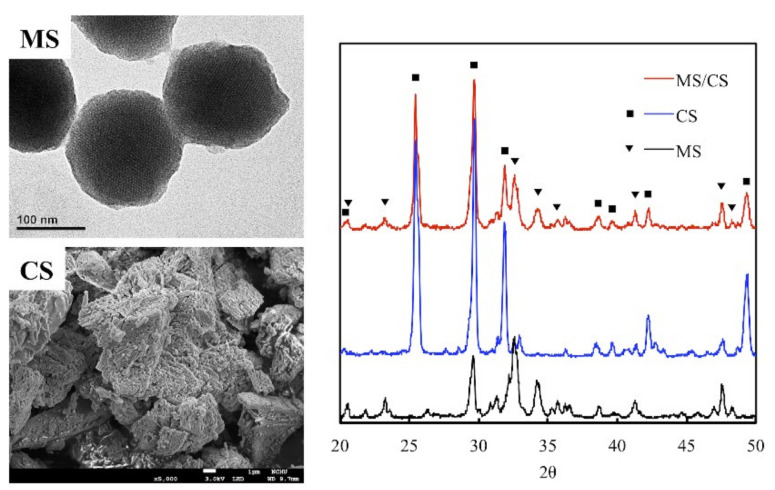
The microstructure and composition of mesoporous calcium silicate (MS) and calcium sulfate (CS) powders and composites. The scale bar of the SEM photograph is 1 µm.

Mesoporous materials scaffolds have been identified as having favorable biocompatible properties that make them suitable for use as bone graft substitutes or drug carriers for bone repair. Studies have proven their efficacy in both in vitro and in vivo studies [28,29]. MS, CS, and MS/CS scaffolds were fabricated with 3D printing according to methods that have been previously reported [17]. Analysis of the morphology has revealed oval-shaped MS nano-particles measuring approximately 150 nm in diameter with multiple porous internal structures that enhance the loaded efficiency of the growth factor (Figure 1). CS displayed a dense and blocky microstructure and a more poorly interconnected pore structure with an overall structure size of approximately 9.7 µm that provided support as the primary source of strength for the scaffold after hydration. X-ray diffractometry (XRD) results showed the highest diffraction peaks at 2*θ* = 26°, 29°, and 32.5° in MS, which corresponds with reflections in the crystalline β-dicalcium silicate (β-Ca2SiO4) [29]. We have previously demonstrated that an MS-based scaffold is characterized by high biocompatibility and osteoinductive qualities and that its open, interconnected pores assist with bone and tissue regeneration [17]. Numerous studies have been published regarding the porosity of scaffolds, rough scaffold surfaces, and their relationship with enhanced bone healing [17,30]. Furthermore, calcium silicate/chitosan or CS combined with a mesoporous scaffold have been shown to improve bone regeneration [31].

### 3.2. In Vitro Bioactivity

**Figure 2:**
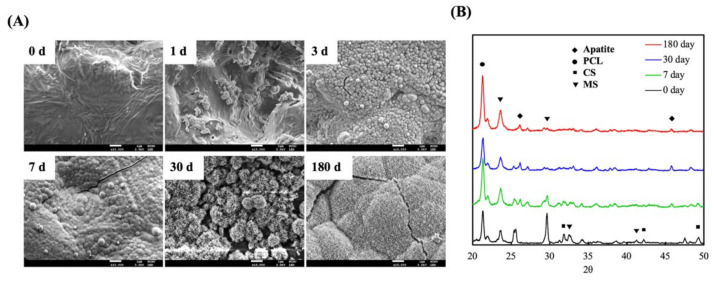
(**A**) Field emission–scanning electron microscopy (FE-SEM) photographs and (**B**) X-ray diffractometry (XRD) of an MS/CS scaffold after soaking in SBF. The scale bar of the SEM photograph is 1 µm.

Apatite formation ability is usually used to predict in vivo bone bioactivity of scaffolds [32]. To confirm bioactivity, we observed apatite precipitation on the surfaces of the scaffolds over a specific time period. The surface morphology and microstructure of the MS/CS scaffold were characterized with SEM and XRD after it was immersed in SBF. The MS/CS scaffold exhibited a smooth and flat surface before being immersed in SBF (Figure 2). However, the composition contains 50% MS/CS nano-particles, which cannot be found on the surface microstructure. It is speculated that during the printing process of the scaffold, the composite material in the syringe is pushed through the needle by air pressure so that the nano-particles on the surface are squeezed into the scaffold. After 1 and 3 days of immersion in SBF, apatite precipitation was observed on the surface of the MS/CS scaffold, with spherical aggregated minerals with an average diameter of approximately 1 μm. Notably, the apatite aggregates generally covered the surface of the scaffold. After 180 days, the surface was completely covered with high-density, needle-shaped crystals and cracks. Previous studies have shown that 3D printing for the treatment of bone disease is a convenient process that can help to hasten healing after surgery [22]. Calcium-silicate-based materials have been identified as causing hydroxyapatite precipitation and crystallization, which substantially enhance bone formation in bone defect regions [22]. A combination of collagen and hydroxyapatite or β-tricalcium phosphate in scaffolds made of polymer biomaterials was found to activate the bone formation signaling pathway, which subsequently led to bone regeneration [33]. One study showed that after immersion in SBF for 7 days, the surface of a mesoporous calcium magnesium silicate scaffold contained nano-pore particles when viewed under high magnification. Another study showed that MC3T3-E1 cells cultured with mesoporous bioactive glass scaffolds for a week adhered strongly to the surface of the scaffolds and were reported to have induced bone mineralization after 3 weeks of culture [34].

Figure 2B shows the MS/CS scaffold after immersion in SBF for different periods of time. We observed a diffraction peak at 2*θ* = 25.9° and between 31.8 and 32.9°, which corresponded with apatite crystals. After 180 days of immersion, the hydroxyapatite (HA)-induced peaks were more apparent than those that appeared after other periods of immersion. The phosphorus ion is a native component of an in-vitro-simulated body fluid or solution and thus is not considered to be a prerequisite for apatite formation. However, it should be noted that both Ca and P are involved in apatite formation. It was reported in a previous study that poly(lactic-co-glycolic acid) (PLGA) combined with a nano-hydroxyapatite nano-fiber scaffold (PLGA-nHA) induces cellular adhesion, proliferation, and osteogenesis due to a higher release of calcium ions [35]. A synergistic effect has also been observed for Si ions, which serve as efficient apatite nucleators. Ca ions serve as HA precipitation accelerators, thus causing HA precipitation [36]. Our XRD results demonstrate that the MS/CS scaffold has good in vitro bioactivity and biocompatibility.

### 3.3. Degradation and Mechanical Properties of the MS/CS Scaffold

**Figure 3:**
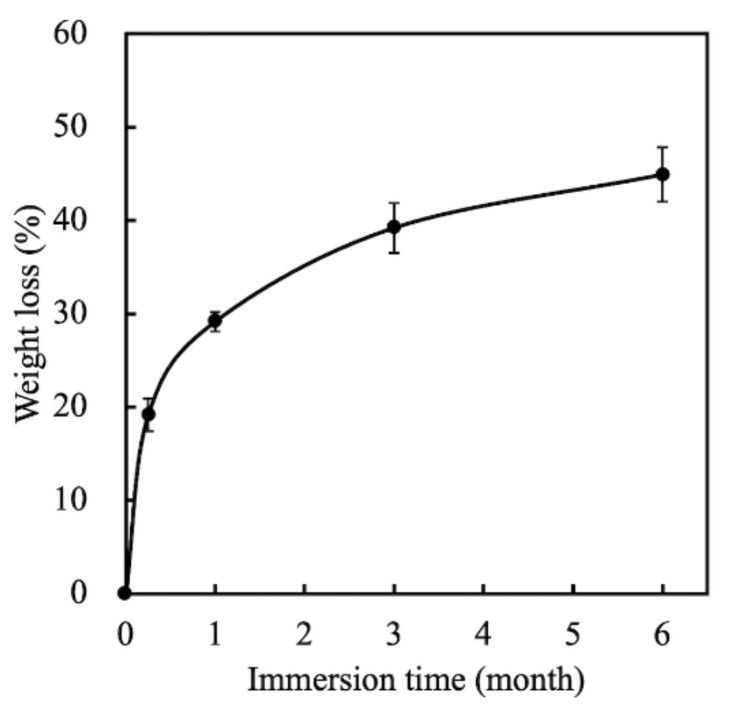
The weight loss of MS/CS scaffolds after soaking in SBF for various time periods.

**Figure 4:**
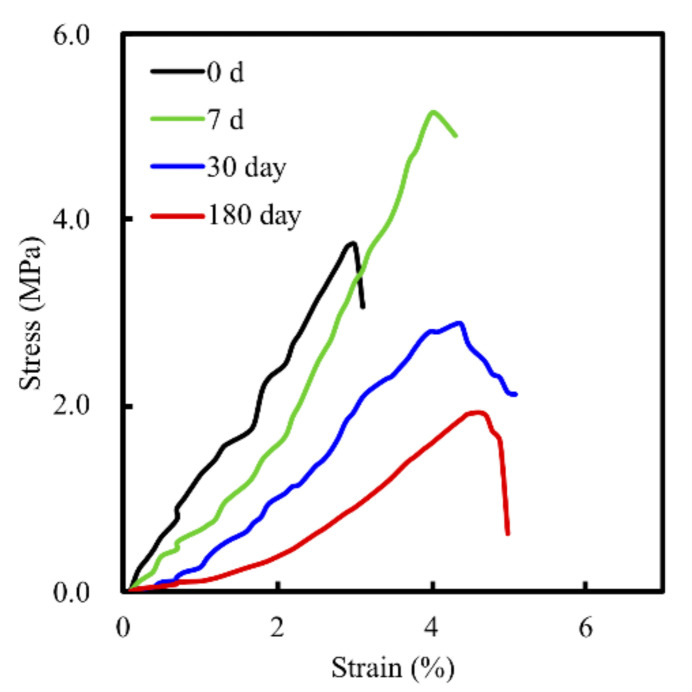
Stress–strain curve of the MS/CS scaffold after immersion in SBF for 7, 30, and 180 days.

Degradation and the mechanical properties of bioscaffolds are considered to be important factors related to bone tissue reconstruction [29]. We measured weight changes in the MS/CS scaffolds soaked in SBF at different time points over a period of 6 months to evaluate them for degradability. According to the weight loss curve, the scaffolds lost a total of approximately 45% of their weight after 6 months (Figure 3). Weight loss was most rapid during the first month, with the scaffolds losing 29.17% of their weight. The rate of weight loss decreased gradually, with the scaffolds losing 39.2% of their weight after 3 months. Notably, the degradability of the MS scaffold remained stable after 6 months of immersion. It was hypothesized that the rapid release during the first month of immersion was due to the higher efficacy of the release of calcium sulfate. As the MS/CS scaffold degrades, Ca and Si ions in the scaffold are released into the surrounding environment, of which Si ions are known to enhance the growth and differentiation of bone cells [37]. The results for the MS/CS scaffold stress–strain test are shown in Figure 4. It can be seen that the MS/CS scaffold gained strength rapidly during the immersion period, where within 7 days, MS/CS scaffolds were found to be 1.3-fold stronger than the control group (3.9 MPa to 5.1 MPa). This result gives rise to the speculation that during the immersion period, the MS/CS ceramic powder came in contact with SBF and started the hydration process and enhanced the yield strength of the scaffolds [38]. However, between 30 and 180 days, the compressive strength values fell significantly in both groups to 2.8 and 1.9 MPa, respectively (*p* < 0.05). At 180 days, the MS/CS scaffold exhibited a 2.6-fold loss in strength and a 1.5-fold loss in weight from the corresponding values recorded at 7 days. However, Young’s moduli of the MS/CS scaffolds were 124.4 ± 8.5, 94.4 ± 4.2, 61.5 ± 5.1, and 46.7 ± 55.8 after immersion in SBF for 0, 7, 30, and 180 days, respectively. The results indicated a decrease in the stiffness of the scaffolds after immersion that was similar to active biodegradation over time [39,40]. Previous research has discussed a correlation between the degradation and mechanical properties of 3D-plotted β-tricalcium phosphate scaffolds and cellular biological functions, including proliferation, adhesion, differentiation, and even in vitro regeneration [36]. The results are shown in Figure 4 and demonstrated that the MS/CS scaffold had good in vitro bioactivity, as seen with the initial increases in the mechanical properties during immersion. Therefore, the MS/CS scaffold mimics the physicomechanical properties of cancellous bone, which has been identified as an effective method to build a bone tissue regeneration scaffold [41,42].

### 3.4. BMP-2 Release

**Figure 5:**
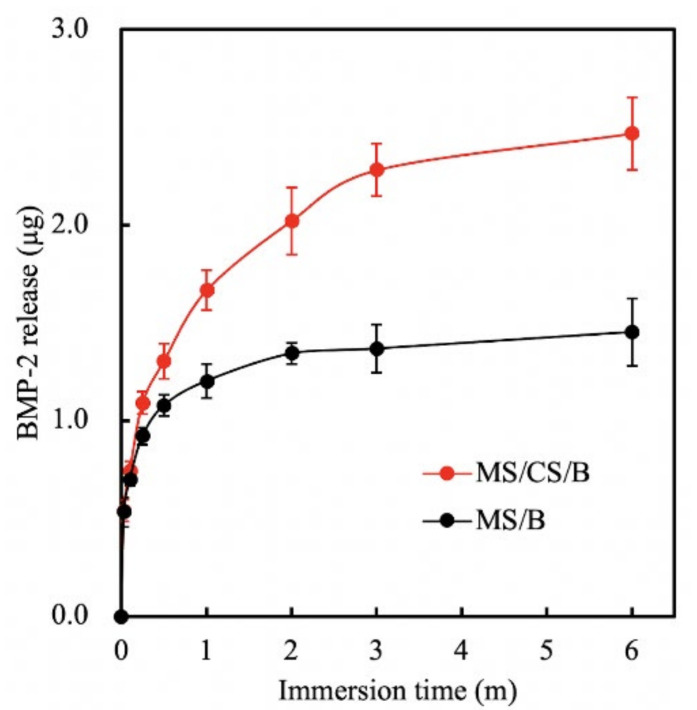
Bone morphogenetic protein-2 (BMP-2) release from an MS/CS/B and MS/B scaffold after immersion in Dulbecco’s modified Eagle’s medium (DMEM) at 37 °C for 6 months.

The mesoporous characteristics of our scaffold meant that growth factors could be conveyed and released more efficiently, thus effectively contributing more toward osteogenesis, especially when compared to nonporous bone scaffolds. Figure 5 shows the cumulative amount of BMP-2 released from drug-loaded MS/CS scaffolds over 6 months. The levels of BMP-2 released from an MS/CS and a bare MS scaffold at 1 month were 1.67 and 1.20 μg, respectively. At 6 months, the levels of released BMP-2 in the MS/CS scaffold group increased to 2.47 μg. The BMP-2 release profiles were proportional to the time durations. During the past few years, nano-porous bioscaffolds have been proven to be good methods for drug delivery and for controlled, sustained release of drug molecules. According to one report, a mesoporous scaffold was loaded with insulin-like growth factor 1 to induce cardiac stem cell proliferation and migration and it was shown to be a potential candidate for in vivo treatment [43]. To investigate whether a higher BMP-2 concentration ratio would increase bone regeneration, we used 3D printing to make nano-pore-sized scaffolds intended to provide a controlled release rate over a 6-month time period. Our results revealed that BMP-2 was continuously released over the immersion period and the concentrations of BMP-2 were directly proportionate to the immersion time. This suggests that the MS/CS scaffold has a high potential for bone repair and could play a vital role in tissue engineering.

### 3.5. Proliferation of hDPSCs on the MS/CS Scaffold

**Figure 6:**
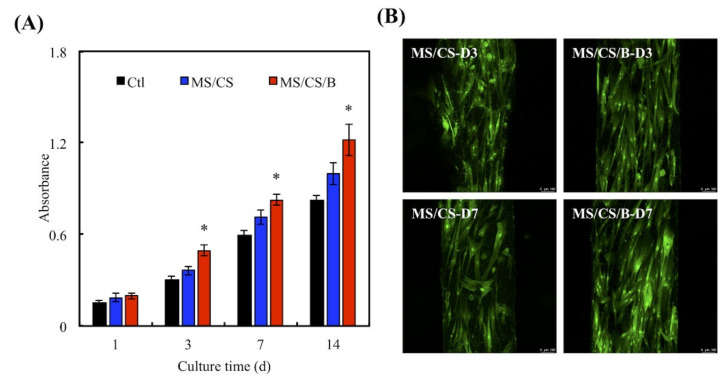
(**A**) The proliferation of the human dental pulp cells (hDPSCs) cultured on scaffolds for 1, 3, 7, and 14 days. * indicates a significant difference (*p* < 0.05) when compared to MS/CS. (**B**) F-actin filament (green) staining of hDPSCs cultured on different scaffolds for 3 and 7 days. The scale bar of the photograph is 100 µm.

In view of evidence indicating that BMP-2 is involved in bone functions such as osteoinduction, osteoconduction, bone metabolism, and osteogenesis [44], we immersed MS/CS scaffolds into BMP-2 solution (0.5 µg/mL) for 12 h to produce MS/CS/BMP-2 (MS/CS/B) scaffolds. For 6 months, hDPSCs were incubated for varying intervals of time on MS/CS/B and MS/CS scaffolds using a controlled drug delivery system. ELISA analysis revealed that the MS/CS/B scaffolds were associated with significantly greater rates of hDPSC proliferation on days 3, 7, and 14 in comparison with both the MS/CS scaffolds and the controlled drug delivery system (Figure 6A). Using fluorescence microscopy of both live and dead cells, we found evidence of hDPSC adherence to the MS/CS/B scaffolds and the growth of nano-pores on the MS/CS/B and MS/CS scaffolds (Figure 6B). Of all three study groups, the MS/CS/B scaffolds were associated with the highest amount of fluorescence on day 7. Compared with the MS/CS scaffolds, the MS/CS/B scaffolds showed significantly greater proliferation and survival potential over time. The assay findings showed that MS/CS/B scaffolds are a suitable delivery system for growth factors.

### 3.6. Western Blot

**Figure 7:**
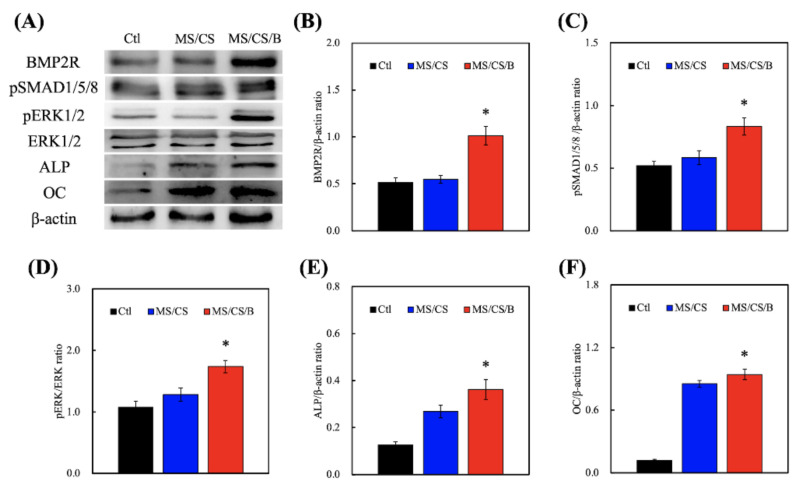
(**A**) Immunodetection of anti-bone morphogenetic protein receptor type II (BMP2R), anti-extracellular signal-regulated kinase 1/2 (ERK 1/2), anti-phospho extracellular signal-regulated kinase 1/2 (p-ERK 1/2), alkaline phosphatase (ALP), osteocalcin (OC), and β-actin protein expression in hDPSCs cultured on scaffolds for 3 days. Quantification of (**B**) BMP2R, (**C**) anti-phospho SMAD1/5/8 (p-SMAD1/5/8), (**D**) p-ERK 1/2, (**E**) ALP, and (**F**) OC. * indicates a significant difference (*p* < 0.05) when compared to MS/CS.

hDPSCs were used to verify whether the MS/CS/B scaffold can effectively induce bone formation and regeneration. The Western blot results quantified BMP2R, p-SMAD1/5/8, p-ERK, ALP, and OC expression on all of the scaffolds (Figure 7). The levels of BMP2R, SMAD1/5/8 phosphorylation, and ERK phosphorylation were significantly increased on the MS/CS/B scaffold, whereas there were no significant incremental increases seen in the control or MS/CS scaffolds. Interestingly, the ALP and OC expression levels were similar for the MS/CS and MS/CS/B scaffolds, indicating that the mesoporous characteristics associated with both scaffolds do promote bone mineralization, osteogenesis, and cell growth. The ALP and OC expression levels, as well as BMP2R and p-ERK expression, were 2-fold and 2.3-fold higher for the MS/CS/B scaffold as compared to the MS/CS scaffold and control delivery system, respectively, while the ALP and OC expression levels were 2.5 times higher for both the MS/CS and MS/CS/B scaffolds as compared to the control delivery system. BMP2R plays a crucial role in mediating the osteogenesis of osteoblast cells, cell differentiation, and bone regeneration [45]. In addition, the activation of intracellular SMAD is known to regulate stem-cell-differentiation-related protein expression that is sensitive to the cultured microenvironment around the cells [46]. A previous study indicated that BMP-2 could stimulate SMAD1/5/8 and affect the osteogenic pathway [26]. Thus, the combined effect of Si ion and BMP-2 could active multiple molecular pathways in several primary cells, resulting in promoted osteogenic differentiation.

### 3.7. Effect of BMPR2 on Cell Proliferation and Differentiation

**Figure 8:**
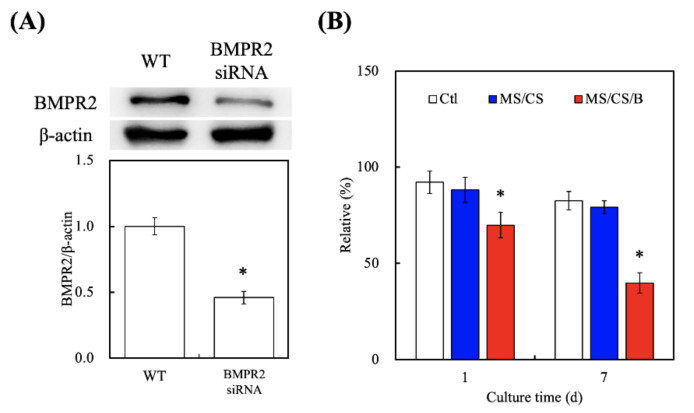
(**A**) Western blotting of knockdown by pairs of BMPR2-specific siRNAs in hDPSCs. (**B**) The effects on siRNA–BMPR2 transfection of proliferation in hDPSCs cultured on scaffolds for 1 and (**B**) 7 days. * indicates a significant difference (*p* < 0.05) when compared to MS/CS.

Figure 8A shows the successful knockdown of BMPR2 using the siRNA as compared to the wide-type group. BMPR2 protein levels were subsequently inhibited by 54% after BMPR2 knockdown as compared to the wide-type group. After BMPR2 knockdown in the MS/CS/B scaffolds, cell quantities were found to be significantly decreased by 21.7% and 49.7% after culture for 1 and 7 days, respectively (Figure 8B). A number of studies on BMP-2 have revealed that BMP-2 is an important mitogen for primary cell behavior and further regulates downstream osteogenic differentiation via the BMP-2/ BMPR2/ mitogen-activated protein kinase (MAPK) signaling pathway [47]. Furthermore, BMP-2 and BMPR2 were trialed in clinical applications for bone fracture regeneration, and local recombinant human BMP-2 was also reported to improve healing in humans [48]. BMPR2 plays an important role in several types of cell migration and proliferation, and many physiologic behaviors of BMPR2 are completed by activating the MAPK and phosphoinositide 3-kinases (PI3K) signal pathways [49,50].

**Figure 9:**
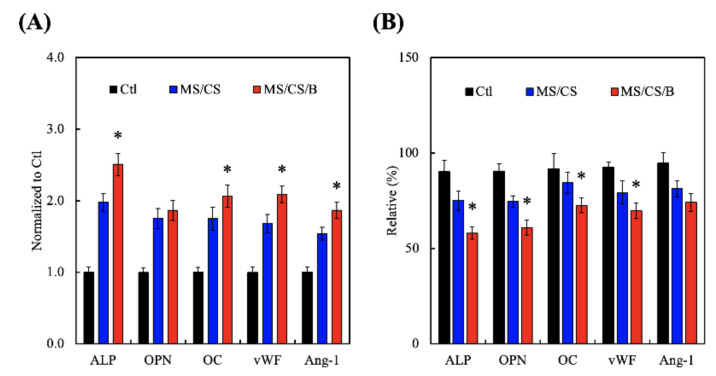
The osteogenic and angiogenic protein expression of (**A**) the hDPSCs and (**B**) the siRNA–BMPR2 transfected hDPSCs on scaffolds for 7 days. * indicates a significant difference (*p* < 0.05) when compared to MS/CS.

Cytokine-carrying scaffolds promote the secretion of bone-related proteins, such as TGF-β, BMP-2, Runt-related transcription factor 2 (Runx2), and osterix, during the bone-healing process. To characterize the role of MS/CS in osteogenesis, we analyzed the in vitro expression profiles of alkaline phosphatase (ALP) and OPN and used ELISA to examine the effectiveness of MS/CS/B in the induction of bone-related proteins and angiogenesis factors (Figure 8A). The levels of ALP, OPN, OC, vWF, and Ang-1 were significantly higher in the hDPSCs from the MS/CS/B scaffold as compared to the cells from the MS/CS scaffold or the control drug delivery system (Figure 9A). Protein expression was higher in the MS/CS/B scaffold than in either of the other two study groups. Vascular endothelial growth factor (VEGF) plays a critical role in cellular survival during the bone regeneration process, and blood supply plays an important role in endochondral ossification, during which vessels sprout and provide nutrients and oxygen that enable bone cells to stimulate cartilage formation and mineralization [51]. Inhibiting VEGF results in bone cell apoptosis, lowers bone mass, and leads to bone fractures [52]. Furthermore, blocking ALP and bone sialoprotein (BSP) interferes with calvarial bone repair, while upregulation of BMP2R, extracellular regulated protein kinases (ERK), ALP, and osteocalcin (OC) bone turnover markers mediates bone formation signaling pathways. In addition, significant (*p* < 0.05) decreases (49%, 39%, and 27%, respectively) in ALP, OPN, and OC (Figure 9B) protein synthesis were found in BMPR2-knockdown hDPSCs cultured on MS/CS/B scaffolds after 7 days. In this study, the expression of vWF and Ang-1 of hDPSCs proved that angiogenesis has a similar pattern for the BMPR2 signaling pathway of osteogenesis.

### 3.8. Mineralization

**Figure 10:**
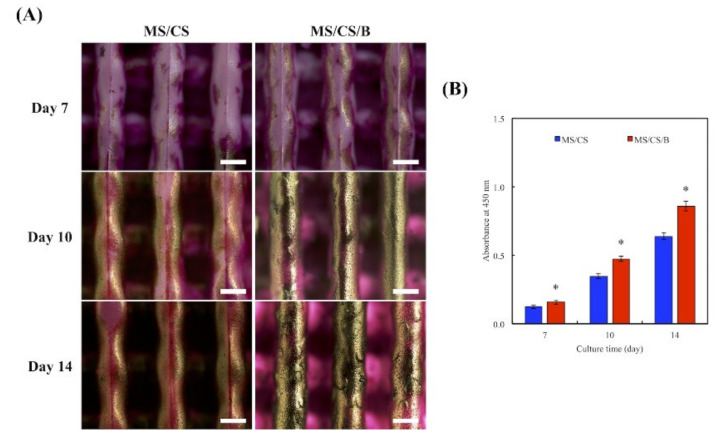
(**A**) Alizarin red S staining and (**B**) quantification of hDPSC calcium mineral deposits cultured on specimens for 7, 10, and 14 days. * indicates a significant difference (*p* < 0.05) when compared to MS/CS. The scale bar of the Optical microscope photograph is 400 µm.

Bone mineralization is an important index for osteogenesis [45]. In this study, hDPSCs were used to confirm in vitro bone cell mineralization of MS/CS/B scaffolds. Alizarin red S staining on the MS/CS/B scaffold revealed efficient mineralization within 2 weeks, with evidence of many mineralized nodules as compared to the MS/CS scaffold (Figure 10A). On the other hand, the ELISA results are depicted in Figure 9B. The results suggested that the calcium sulfate content of the MS/CS/B scaffold accelerated the release of BMP-2 and resulted in higher mineralization formation as compared to the MS/CS scaffold. Our results showed that the MS/CS/B scaffold could modulate bone formation, regeneration, and angiogenesis. This result suggested that it is then essential to further investigate the in vivo role of the MS/CS/B scaffold.

### 3.9. In Vivo Bone Regeneration

**Figure 11:**
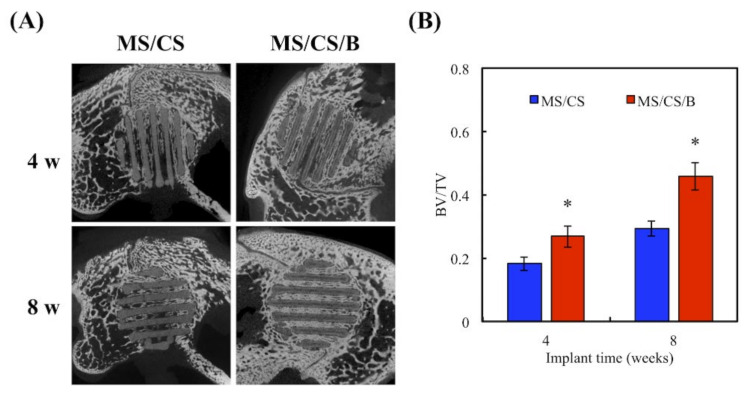
(**A**) Micro-CT image showing the morphology of bone growth for a fixed-sized critical lesion after it underwent 8-week regeneration with scaffolds; (**B**) data analysis of relative bone mass volume (bone volume/tissue volume (BV/TV) ratio) for a fixed-sized critical lesion after it underwent 8 weeks of regeneration with scaffolds. * indicates a significant difference (*p* < 0.05) when compared to MS/CS.

**Figure 12:**
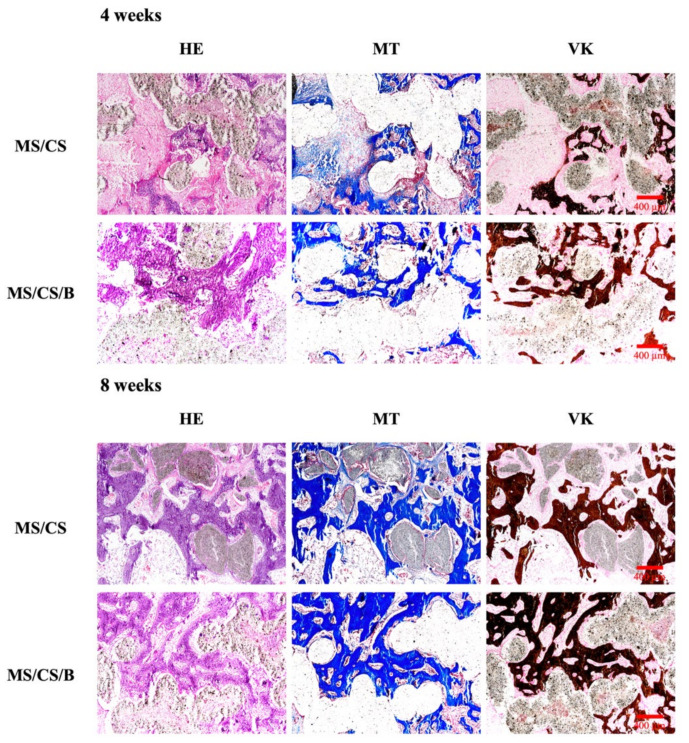
Histological analysis of new bone regeneration around and within the scaffolds in the rabbit femoral defect model. Left, hematoxylin and eosin (HE) stain; middle, Masson’s trichrome (MT) stain; and right, von Kossa (VK) stain of regenerated bone mass after 8 weeks of regeneration in the in vivo experiment. Purple: tissue morphology. Blue: collagen and bone matrix. Brown: bone mineralization. The scale bar of the histological photograph is 400 µm.

After 8 weeks of implantation in rabbit femurs, micro-CT analysis clearly showed time-dependent increments of bone formation, improved bone density, and tissue reconstruction with the MS/CS/B scaffold as compared to the MS/CS scaffold (Figure 11). The MS/CS/B scaffold had excellent osteoinduction characteristics due to the fact that it was surrounded by newly regenerated bone. Simultaneously, with the implantation of the MS/CS/B scaffold, the bone volume/tissue volume (BV/TV) ratio was also upregulated over time. Thus, it can be seen that BMP-2 and osteogenesis are critical factors in bone healing [53,54]. Recently, a 3D-printed ceramic scaffold was shown to have osteoinductive potential, and it was found that the scaffold improved bone cell mineralization in a calvarial defect model. Micro-CT showed that the ceramic scaffold improved tissue repair and cell ingrowth in pores in order to fill the defect region. Therefore, osteoinduction and tissue reconstruction induced by bioscaffold activation are important factors in bone regeneration and bone fracture repair [53,54]. In this study, we applied micro-CT technology to investigate bone regeneration and to quantify the bone volume and tissue volume ratios. Our results demonstrated that the MS/CS/B scaffold could support bone defect healing and provide a good microenvironment in which to accelerate the repair of damaged tissue. The time-dependent mineralization associated with an MS/CS/B scaffold is considered to be important in the treatment of bone diseases. Histologic analysis of the femur revealed that the MS/CS/B scaffold had markedly increased bone tissue structure, collagen formation, osteoid tissue, and greater proportions of calcified bone. The above phenomena were further confirmed via H&E, Masson’s trichrome (MT), and von Kossa staining (VK) (Figure 12), thereby suggesting that the MS/CS/B scaffold was able to effectively increase in vivo bone regeneration and bone formation. Consistent with the micro-CT results, H&E staining showed more tibia integrity in the MS/CS scaffold groups with or without BMP-2. MT and VK staining also indicated collagen and calcification were upregulated via the MS/CS/B scaffold 8 weeks after implantation. Taken together, our findings demonstrated that the 3D-printed, radially oriented MS/CS/B scaffold is significant in bone regeneration in the rabbit tibia defect model. Previously, Li et al. suggested that different kinds of mesoporous bioactive ceramic scaffolds may contribute to osteogenesis and chondrogenesis in a clinical setting [55]. The excellent osteoconduction in ceramic-based scaffolds due to the ceramic materials promoted the bone formation and also led to bone repair in ovine tibia defect patients [56]. In addition to ceramic-material-induced bone repair, other research has reported that growth factor BMP-2 treatment increases bone formation in bone defects within the first few weeks, thus indicating that BMP-2 may play a role in mediating bone activity in osteogenesis and bone formation [57]. Increasing evidence indicates that upregulation of collagen and mineralization contributes significantly to bone development and bone homeostasis [29]. Our study showed that the MS/CS/B scaffolds were able to increase cell proliferation, osteogenesis, and angiogenesis, thereby indicating their potential in the treatment of bone disorders or bone loss. In conclusion, the experimental evidence from this study suggested that the MS/CS/B scaffold had a high degradation rate and high mechanical strength and was able to enhance ALP, OPN, OC, vWF, and Ang-1 expression in a time-dependent manner. The in vivo evidence demonstrated that the MS/CS/B scaffold had excellent osteoinduction and osteogenesis characteristics. 

## 4. Conclusions

In this study, bioactive MS was synthesized by a sol-gel method using the surfactant CTAB as a template and was successfully printed onto 3D scaffolds by combining MS/CS and PCL. The 3D-printed scaffold had even, porous structures and retained its bioactive qualities when immersed in SBF solution. It had an overall degradation of 44.91% over a 6-month period and was accompanied by corresponding rates of degradation and decreases in compressive strength. When preloaded with BMP-2 and VEGF, the MS/CS/B scaffold was associated with prolonged, controlled, gradual drug release over 6 months. Cellular behavior studies showed that BMP-2 loading promoted osteogenic responses of hDPSCs due to upregulation of other bone-related genes as well as higher mineralization rates when compared to the control group. The formation of new bone ingrowth into the scaffold was observed in computed tomography images and histological findings. These results suggest that 3D-printed MS/CS scaffolds containing BMP-2 show potential for use in hard tissue engineering. Significantly, we set a high-efficacy platform to realize cell biological functions in vitro and in vivo. We will add other potential parameters, such as porosity and drug release, in the long term and will use this model to develop various bone and vascular substitute scaffolds. To sum up, we provided a novel insight into a multifunctioning 3D-printed mesoporous scaffold and advocate it as a prospective candidate to augment bone healing.

## Data Availability

Data available in a publicly accessible repository.
